# Plasma Antimicrobial Peptide LL-37 Level Is Inversely Associated with HDL Cholesterol Level in Patients with Type 2 Diabetes Mellitus

**DOI:** 10.1155/2014/703696

**Published:** 2014-03-26

**Authors:** Shu Meguro, Masuomi Tomita, Takeshi Katsuki, Kiyoe Kato, Henpiru Oh, Akira Ainai, Ryo Ito, Toshihide Kawai, Hiroshi Itoh, Hideki Hasegawa

**Affiliations:** ^1^Division of Endocrinology, Metabolism and Nephrology, Department of Internal Medicine, School of Medicine, Keio University, 35 Shinanomachi, Shinjuku-ku, Tokyo 160-8582, Japan; ^2^Department of Internal Medicine, Saiseikai Central Hospital, 1-4-17 Mita, Minato-ku, Tokyo 108-0073, Japan; ^3^Minami-Aoyama Home Clinic, 7-5-2 Minami Aoyama, Minato-ku, Tokyo 107-0062, Japan; ^4^Department of Pathology, National Institute of Infectious Disease, 1-23-1 Toyama, Shinjuku-ku, Tokyo 162-0052, Japan

## Abstract

*Introduction*. Relation between atherosclerosis and innate immunity has attracted attention. As the antimicrobial peptide, LL-37, could have an important role in atherosclerosis, we supposed that there could be a meaningful association of plasma LL-37 level with risk factors for cardiovascular disease in subjects with type 2 diabetes mellitus. *Materials and Methods*. We evaluated plasma LL-37 level and other clinical markers in Japanese subjects with type 2 diabetes mellitus (*n* = 133, 115 men and 18 women; age 64.7 ± 11.5 years; HbA1c 8.1 ± 1.6%). Plasma level of LL-37 was measured by ELISA. *Results*. Mean plasma LL-37 level was 71.2 ± 22.3 ng/mL. Plasma LL-37 level showed significant correlations with HDL cholesterol (*r* = −0.450, *P* < 0.01), triglyceride (*r* = 0.445, *P* < 0.01), and high sensitive C-reactive protein (*r* = 0.316, *P* < 0.01) but no significant correlation with age, body mass index, HbA1c, estimated glomerular filtration rate, 25-hydroxyvitamin D, or vitamin D binding protein. Multiple linear regression analysis showed significant correlations of plasma LL-37 level with HDL cholesterol (*β* = −0.411, *P* < 0.01) and high sensitive C-reactive protein (*β* = 0.193, *P* < 0.05). *Conclusion*. Plasma LL-37 level was positively correlated with inflammatory markers and negatively correlated with HDL cholesterol in patients with type 2 diabetes mellitus.

## 1. Introduction

Antimicrobial peptides are peptides that can kill viruses, fungi, bacteria, and other microbes [1]. They are produced in most multicellular organisms and expressed on epithelial surfaces and within circulating white cells, constitutively or in response to stimuli such as tissue injury, through interleukin-1 and other cytokines or microbial components such as lipopolysaccharide (LPS). Among such peptides, which play important roles in the innate immune system, LL-37, also known as cathelicidin, is one of the most studied. In addition to its anti-infective activities, LL-37 stimulates local angiogenesis, acts synergistically with the epidermal growth factor receptor to promote epithelial growth, and attracts monocytes and neutrophils through formyl peptide receptors on these cells. In this way the peptide helps orchestrate the inflammatory process [2–4].

Innate immunity is important for primary defense not only against antimicrobial infections, but also against atherosclerosis via nonspecific inflammatory processes [5]. Involvement of LL-37 in the atherosclerotic process was suggested by previous reports. Increased transcription of LL-37 was demonstrated in human atherosclerotic plaques [6], and the presence of LL-37 in human atherosclerotic lesions obtained at autopsy was shown by immunohistochemical study and it induced death of vascular smooth muscle cells [7]. A recent study in a rodent model showed that cathelicidin-related antimicrobial peptide (CRAMP: mouse cathelicidin equivalent to LL-37 in human) and Apo E double knockout mice exhibited reduced atherosclerotic lesions compared to Apo E knockout mice [8].

Type 2 diabetes mellitus is a worldwide epidemic and increases macrovascular and microvascular risk [9, 10]. Residual risk for cardiovascular disease still exists in spite of widely adopted statin treatment. As one of the explanations for the residual risk of atherosclerosis, chronic subclinical inflammation is a candidate [11]. We therefore suppose that analysis of plasma LL-37 level, which is an important player in the innate immune system, in subjects with type 2 diabetes mellitus may be meaningful in regard to its association with risk factors for cardiovascular disease.

## 2. Materials and Methods

The present study was conducted according to the principles expressed in the Declaration of Helsinki. Written informed consent was obtained from each subject after a full explanation of the purpose, nature, and risk of all procedures used. The protocol was approved by the ethical review committees of Saiseikai Central Hospital, Tokyo, Japan.

We originally aimed this study to observe the association between pandemic influenza virus A/H1N1pdm09 infection and plasma 25-hydroxyvitamin D [25(OH)D] and/or plasma LL-37 level in subjects with type 2 diabetes mellitus who were considered as high risk for influenza infection. Subjects were recruited in routine outpatient clinics for their diabetes managements. Patients with hematological disorders, liver cirrhosis, malignant disease, or active gastrointestinal disease were not enrolled in this study. Patients with active inflammatory or infectious disease were excluded by their comorbidities, clinical symptoms, and body temperatures. As plasma 25(OH)D level is influenced by their renal function, we actively enrolled subjects with nephropathy to obtain equivalent numbers of subjects with each clinical stage of diabetic nephropathy according to their laboratory data at the consent to evaluate the effect of advanced stages of diabetic nephropathy. As a result, we measured plasma LL-37, 25(OH)D, vitamin D binding protein (VDBP), and other clinical markers in Japanese subjects with type 2 diabetes mellitus (*n* = 135, 117 men and 18 women; age 64.7 ± 11.5 years; HbA1c 8.1 ± 1.6%) who attended the outpatient clinic of Saiseikai Central Hospital between September 2009 and February 2010.

After obtaining written informed consent, blood samples in an overnight fasting state were drawn at the next visit to the usual outpatient clinic. Routine blood analysis was performed by the hospital laboratory immediately after blood sampling. After centrifugation, a part of the plasma was preserved at −80°C until further analysis. Assays of fasting plasma glucose (FPG), total cholesterol, HDL cholesterol, triglyceride, serum creatinine level, and several other biochemical parameters were performed with autoanalyzers. HbA1c level was determined by high-performance liquid chromatography (Arkray Inc., Kyoto, Japan) according to the recommended method by the Japan Diabetes Society at that time and converted to the National Glycohemoglobin Standardization Program (NGSP) value [12]. Microalbuminuria was defined as an albumin creatinine ratio (ACR) of 30 to 300 mg/g Cr and macroalbuminuria as ACR of more than 300 mg/g Cr in spot urinalysis. Estimated glomerular filtration rate (eGFR) (mL/min/1.73 m^2^) was calculated using the equation provided by the Japanese Society of Nephrology as 194 × Cr^−1.094^ × Age^−0.287^ (with further multiplication by 0.739 for female subjects) [13]. Plasma levels of LL-37, 25(OH)D, and VDBP were measured by ELISA using a human LL-37 ELISA kit (Hycult Biotechnology, Uden, Netherlands), 25(OH)-vitamin D direct ELISA kit (Immundiagnostik AG, Bensheim, Germany), and vitamin D binding protein ELISA kit (Immundiagnostik AG, Bensheim, Germany), respectively, according to the manufacturers' manuals, at the laboratory of the National Institute of Infectious Diseases.

Hypertension was defined as systolic blood pressure >140 mmHg, diastolic blood pressure >90 mmHg, or the prescription of antihypertensive medication. Dyslipidemia was defined as LDL cholesterol >3.63 mmol/L, triglyceride >1.72 mmol/L, HDL cholesterol <1.04 mmol/L, or the prescription of lipid-lowering medication. All subjects underwent funduscopic examination by trained ophthalmologists. Clinical stage of diabetic retinopathy was classified based on the Davis classification as none, background retinopathy, and more advanced stage or previous history of photocoagulation [14]. Clinical stage of diabetic nephropathy was classified as none, microalbuminuria, macroalbuminuria, chronic renal failure (CRF), which was defined as eGFR less than 30 mL/min/1.73 m^2^, and dialysis. Past history of cardiovascular disease was determined by checking the medical records or detailed medical interview. Prior history of myocardial infarction, coronary intervention, ischemic stroke, or peripheral artery disease was regarded as a history of CVD.

Continuous variables are expressed as mean ± SD. Correlation coefficients were analyzed by Pearson's test. However, triglyceride and high sensitive C-reactive protein (hs-CRP) were analyzed by Spearman's rank test because of their skewed distributions. Differences in continuous variables between the two groups were tested by Student's *t*-test, and differences among three or more groups were tested by analysis of variance (ANOVA). Multiple linear regression analysis with compulsory input was performed to evaluate the independent contribution to plasma LL-37 level. Age, sex, BMI, disease duration, hypertension, HbA1c, HDL cholesterol, and hs-CRP were adopted as covariates. Values of *P* < 0.05 were considered statistically significant. All analyses were performed using SPSS 21.0 statistical software (SPSS Inc., Chicago, Il, USA).

## 3. Results

Details of the patients' characteristics are shown in Table 1. Among them, 65.4% were prescribed renin-angiotensin system (RAS) blockade medication, 57.9% were prescribed a statin, and 55.6% were treated with insulin. No one revealed the signs of influenza virus infection during that season.

**Figure 1:**
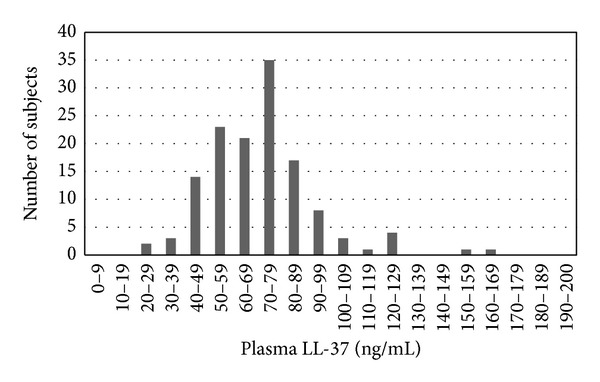
Histogram of frequency distribution of plasma LL-37 level.

**Figure 2:**
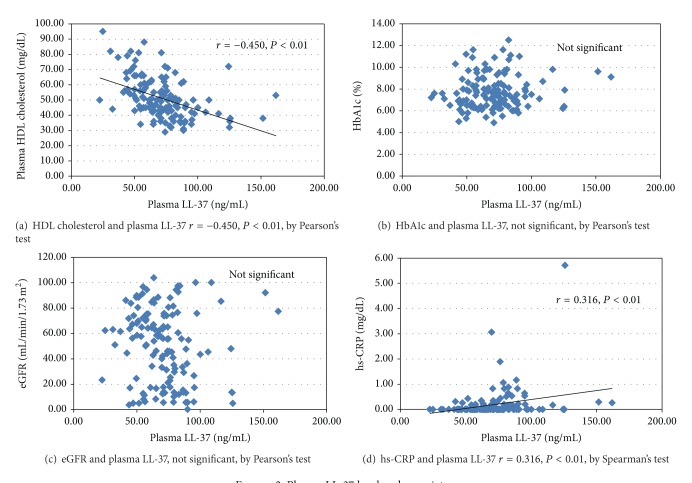
Plasma LL-37 level and covariates.

Plasma LL-37 level was 71.2 ± 22.3 ng/mL (mean ± SD) and showed an almost normal distribution (Figure 1). Plasma LL-37 level showed significant correlations with HDL cholesterol (*r* = −0.450, *P* < 0.01), triglyceride (*r* = 0.445, *P* < 0.01), and hs-CRP (*r* = 0.316, *P* < 0.01), but no significant correlation with age, disease duration, BMI, HbA1c, eGFR, 25(OH)D, or VDBP (Table 2, Figure 2).

Plasma LL-37 level was not different between genders. The presence of hypertension and smoking status showed no correlation with plasma LL-37 level, but the presence of dyslipidemia showed a significant correlation with plasma LL-37 level (*P* < 0.01). The clinical stage of diabetic retinopathy did not show a significant correlation with plasma LL-37 level. The clinical stage of diabetic nephropathy did not show a tendency for a relation with LL-37 level. Plasma LL-37 level did not differ according to history of cardiovascular disease. Treatment with a RAS blocker, statin, or insulin did not show a significant difference between the groups either. HDL cholesterol level did not differ according to history of cardiovascular disease, and statins were frequently prescribed in subjects with a history of cardiovascular disease (chi squared analysis, *P* < 0.01).

Multiple linear regression analysis showed significant correlations of plasma LL-37 level with HDL cholesterol (*β* = −0.411, *P* < 0.01) and hsCRP (*β* = 0.193, *P* < 0.05) (Table 3).

## 4. Discussion

This study demonstrated that plasma LL-37 level was positively correlated with inflammatory markers and negatively correlated with HDL cholesterol in patients with type 2 diabetes mellitus. These results are very interesting in view of several recent reports that elucidated an association between innate immunity and atherosclerosis.

There was a gender disproportion in this study subjects although there was not a statistical difference in plasma LL-37 level between genders. This occurred unintentionally in our recruiting process. It was partly because of the patient population of our hospital (more than two-third were male). Also, although we aimed to obtain equivalent numbers of subjects with each clinical stage of diabetic nephropathy, the number of the subjects without microalbuminuria was smaller than the other stages of nephropathy. This occurred by the visit to visit variation of ACR values. As the plasma level of LL-37 was not different between genders and among the clinical stages of nephropathy, we did not consider that this disproportion of the study population affected the results.

Type 2 diabetes mellitus is a major risk factor for cardiovascular disease [15, 16]. Reduced HDL cholesterol and increased triglyceride are typical features of dyslipidemia in type 2 diabetes mellitus [17]. Although recent large interventional clinical trials in type 2 diabetes mellitus failed to show a reduction in mortality or cardiovascular events [18–20], residual risk for cardiovascular disease still exists in spite of widely adopted statin treatment. Interventions on HDL cholesterol are one of the most promising treatments for residual risk for cardiovascular disease [21, 22]; however, some studies of cholesterol ester transfer protein (CETP) inhibitors, which increase plasma HDL cholesterol level, failed to show a protective effect against cardiovascular disease [23–25]. As a result, the mechanism of the preventive effect of HDL cholesterol on the atherosclerotic process is still being researched [26], even though it is now accepted that a higher HDL cholesterol level is beneficial in terms of cardiovascular disease. In this context, the relation between HDL cholesterol and immunity is receiving a lot of attention [27]. For example, apoprotein A-I, which is one main apoprotein in HDL, has a protective effect from lipopolysaccharide (LPS) toxicity [27]. The lipid composition change of lipid rafts on cell membrane occurs by the cholesterol efflux through the interaction between HDL and lipid rafts. This change seems to modulate the series of immune responses, including monocytes, macrophages, and B and T lymphocytes [28]. HDL also transports several immunologically active substances, such as sphingosine-1-phosphate [27, 28]. We did not evaluate apoprotein fractions in this study so we could not assess if apoprotein composition in HDL affect our results. However, we consider that our results add new evidence for a link between HDL cholesterol and immunity.

Chronic inflammation is involved in the pathogenesis of CVD. Several prospective studies indicated that an elevated hs-CRP level is a risk factor for CVD [29–32]. Randomized control study by the usage of statin also revealed that the achievements of lower levels of both the LDL cholesterol and the hs-CRP are independently related to the incident of CVD [33, 34]. The correlation between hs-CRP and LL-37, which we demonstrated in this study, might suggest the association of innate immune process with the chronic inflammation and elevated hs-CRP level.

As written in Section 1, LL-37 has multifactorial functions such as antimicrobial effect, antiviral effect, as well as induction of chemotaxis and stimulation of cytokine release [1–4]. LL-37 indirectly attracts more immune cells by inducing secretion of IL-8 from macrophages, fibroblasts, and epithelial cells [3]. LL-37 also induces expression of the adhesion molecule intercellular adhesion molecule-1 and the chemokine monocyte chemoattractant protein 1 in endothelial cells and induces the death of smooth muscle cells [6]. This series of phenomenon induced by LL-37 has been considered important in progression and rupture of atheromatous plaque. LL-37 might play a pivotal role in this series of process interactively with HDL. We suppose that the role of LL-37 in atherosclerosis should be studied further.

In this study, plasma LL-37 level was associated with HDL cholesterol level but not with a history of cardiovascular disease or statin treatment. However, statins are frequently prescribed in patients with a history of cardiovascular disease. As plasma HDL cholesterol level was not different regardless of history of cardiovascular disease, subjects with a history might have shown lower HDL cholesterol levels without statin treatment.

It is also known that LL-37 is induced by vitamin D, and the gene encoding LL-37 contains sites for the vitamin D receptor [1]. So the link between vitamin D and innate immunity is also being researched [35, 36]. In our study, plasma 25(OH)D level and VDBP level were not associated with plasma LL-37 level. One of the characteristics of our study subjects is that we included many subjects with advanced stages of diabetic nephropathy, which made mean 25(OH)D level in our study relatively low. There must be a complicated mechanism for the association between vitamin D and LL-37 involving obesity, diabetes, dyslipidemia, renal impairment, and the immune system. We cannot explain the reason for this dissociation, but the characteristics of the study group might have influenced the results.

There are some limitations of this study. One is that it had a cross-sectional design and was performed at one facility, so the results should be confirmed at other facilities in the future. Most subjects in this study had long duration of diabetes and relatively advanced complications. As duration of diabetes did not influence the level of plasma LL-37, we suppose we can expect the same relation in the subjects with shorter duration of disease, but it should be confirmed in the future. As another limitation, the clinical significance of plasma LL-37 level is still not certain. A complicated mechanism might exist in the association between HDL cholesterol and plasma LL-37 level. However, this study showed a correlation between plasma HDL cholesterol level and plasma LL-37 level. Further research is needed to clarify the relation between atherosclerosis and immunity, in order to reduce the residual risk for cardiovascular disease.

## Figures and Tables

**Table 1:** Clinical characteristics of study subjects.

Age (years)	64.7 ± 11.5
Sex (male/female)	115/18
BMI (kg/m^2^)	25.6 ± 4.5
Disease duration (years)	18.3 ± 10.5
Hypertension (%)	85.0
Dyslipidemia (%)	76.7
Smoking (%)	27.1
FPG (mmol/L)	9.6 ± 3.1
HbA1c (%)	8.1 ± 1.6
Total cholesterol (mmol/L)	4.8 ± 0.8
HDL cholesterol (mmol/L)	1.3 ± 0.4
Triglyceride (mmol/L)	1.7 ± 1.0
Plasma creatinine (mmol/L)	183 ± 206
eGFR (mL/min/1.73 m^2^)	50.4 ± 29.1
25(OH)D (ng/mL)	71.2 ± 22.3
VDBP (mg/dL)	31.7 ± 39.1
hs-CRP (mg/dL)	0.19 ± 0.61
WBC (mm^3^)	7091 ± 2058
Retinopathy (none/simple/proliferative)	51/53/29
Nephropathy (none/microalbuminuria/macroalbuminuria/CRF/dialysis)	23/40/31/31/8
History of cardiovascular disease (%)	32.3

Continuous variables are expressed as mean ± SD. FPG: fasting plasma glucose, eGFR: estimated glomerular filtration rate, 25(OH)D: 25-hydroxyvitamin D, VDBP: vitamin D binding protein, hs-CRP: high sensitive C-reactive protein, WBC: white blood cell count, and CRF: chronic renal failure.

**Table 2:** Correlation of plasma LL-37 level with clinical covariates.

	*r*	*P* value
Age	−0.161	n.s.
BMI	0.128	n.s.
Disease duration	−0.165	n.s.
Plasma creatinine	0.070	n.s.
eGFR	−0.098	n.s.
Uric acid	0.082	n.s.
Total cholesterol	0.073	n.s.
HDL cholesterol	−0.450	<0.01
LDL cholesterol	0.132	n.s.
Triglyceride	0.445	<0.01
FPG	0.027	n.s.
HbA1c	0.085	n.s.
hs-CRP	0.316	<0.01
WBC	0.162	n.s.
25(OH)D	−0.164	n.s.
VDBP	0.092	n.s.

eGFR: estimated glomerular filtration rate, FPG: fasting plasma glucose, hs-CRP: high sensitive C reactive protein, WBC: white blood cell count, 25(OH)D: 25-hydroxyvitamin D, and VDBP: vitamin D binding protein. n.s.: not significant.

**Table 3:** Multiple linear regression analysis of plasma LL-37 level.

*R* ^2^	0.283	
Variables	Beta	*P* value
Age	−0.156	n.s.
Sex	−0.131	n.s.
BMI	−0.116	n.s.
Disease duration	−0.124	n.s.
Hypertension	−0.120	n.s.
HbA1c	0.024	n.s.
HDL cholesterol	−0.411	<0.01
hs-CRP	0.193	<0.05

Beta is the standardized partial regression coefficient of multiple linear regression analysis. n.s.: not significant.
